# Atmospheric mercury monitoring data across Italy: A multi-year national dataset (2020 - 2023)

**DOI:** 10.1016/j.dib.2026.112622

**Published:** 2026-02-21

**Authors:** Mariantonia Bencardino, Antonella Tassone, Maria Martino, Francesco D’Amore, Teresa Sprovieri, Carmine Ungaro, Virginia Andreoli, Giulio Esposito, Giorgio Siliprandi, Guido Lanzani, Lorenzo Angiuli, Alessandra Nocioni, Cristina Leonardi, Francesca Sprovieri, Nicola Pirrone

**Affiliations:** a Institute of Atmospheric Pollution Research of the Italian National Research Council (CNR-IIA), c/o UNICAL Polifunzionale 87036 Rende, Italy; b Institute for High Performance Computing and Networking of the Italian National Research Council (CNR-ICAR), Via Pietro Bucci 87036 Rende, Italy; c Institute of Atmospheric Pollution Research of the Italian National Research Council (CNR-IIA), Strada Provinciale 35D 00010 Montelibretti, RM, Italy; d Regional Agency for the Protection of the Environment - Lombardy (ARPA-Lombardia), Viale Risorgimento, 43 46100 Mantova, Italy; e Regional Agency for the Protection of the Environment - Lombardy (ARPA-Lombardia), Via Rosellini 17 20124 Milano, Italy; f Regional Agency for the Protection of the Environment - Apulia (ARPA-Puglia), Corso Trieste 27 70126 Bari, Italy; g Regional Agency for the Protection of the Environment - Apulia (ARPA-Puglia), Via Galanti 16 72100 Brindisi, Italy; h Institute of Atmospheric Pollution Research of the Italian National Research Council (CNR-IIA), c/o MASE, Via C. Colombo 44 00144 Roma, RM, Italy

**Keywords:** Atmospheric monitoring network, European air quality directive, Minamata convention, Environmental policy, Total Gaseous Mercury (TGM), Gaseous Oxidized Mercury (GOM), Particulate-bound Mercury (PBM)

## Abstract

In compliance with the European Air Quality Directive, an Italian monitoring network was established under the framework of the “Reti Speciali” agreement. A key component of this framework is the monitoring of atmospheric mercury (Hg) across the Italian territory.

To this end, a dedicated Hg network was designed including three ground-based monitoring stations, strategically located from Northern to Southern Italy, to identify potential geographical variations.

Atmospheric Hg was measured using automated instrumentation, providing high-resolution time series for Total Gaseous Mercury (TGM) or Gaseous Elemental Mercury (GEM). While all three stations monitored these primary Hg species, one master station provided advanced speciation data, including Gaseous Oxidized Mercury (GOM) and Particulate-bound Mercury (PBM).

The measurements carried out within this network produced a comprehensive four-year dataset (2020–2023), establishing the first preliminary baseline for atmospheric Hg concentrations in Italy. These Hg data are supplemented by concurrent meteorological measurements at each station, enabling multi-parameter analysis.

The provided integrated dataset supports a extensive characterization of atmospheric Hg behaviour across the Italian peninsula, enabling the assessment of emission reduction policies mandated by the Minamata Convention on Mercury.

Specifications Table

**Table:** 

Subject	Earth & Environmental Sciences
Specific subject area	Atmospheric mercury monitoring data collection in Italy.
Type of data	Table (.csv format)
Data collection	Continuous TGM/GEM data were collected using two kinds of automated analysers: Tekran 2537X, based on Cold Vapor Atomic Fluorescence Spectrophotometry, and Lumex RA-915, based on Zeeman Atomic Absorption Spectrometry. Speciation (GOM/PBM) was determined at one station, using integrated Tekran 1130/1135 modules. Collection protocols followed the UNI EN 15852:2010 methods. Data were quality-controlled and managed following the established Global Mercury Observation System (GMOS) Standard Operating Procedures (SOPs). To facilitate reproducibility, raw measurements are provided alongside the final validated hourly-averaged atmospheric Hg data. These are complemented by meteorological parameters, recorded via a co-located automated station and included in the corresponding dataset.
Data source location	Data were collected at three sites across the Italian territory and within the dedicated “Reti Speciali” network. The monitoring stations, their geographical coordinates, altitude, and managing organizations are listed below: • Schivenoglia: (45.02° N, 11.08° E, 16 m a.s.l.), located in the province of Mantua, representative of Northern Italy and managed by the Regional Environmental Protection Agency of Lombardy (ARPA Lombardia); • Montelibretti: (42.11° N, 12.64° E, 30 m a.s.l.), located in the province of Rome, representative of Central Italy and managed by the National Research Council – Institute of Atmospheric Pollution Research (CNR-IIA); • Monte Sant’Angelo: (41.67° N, 15.95° E, 125 m a.s.l.), located in the province of Foggia, representative of Southern Italy and managed by Regional Environmental Protection Agency of Apulia (ARPA Puglia).
Data accessibility	Repository name: ZENODO Data identification number: 10.5281/zenodo.18559721 Direct URL to data: https://doi.org/10.5281/zenodo.18559721
Related research article	Bencardino, M., Tassone, A., Martino, M., D’Amore, F., Sprovieri, T., Ungaro, C., Andreoli, V., Esposito, G., Siliprandi, G., Lanzani, G., Angiuli, L., Nocioni, Leonardi, C., Sprovieri, F. and Pirrone, N. (2025). Establishing a national network for atmospheric mercury monitoring: preliminary spatial and temporal insights from Italy. Atmospheric Environment, 121,477. Doi: 10.1016/j.atmosenv.2025.121477.

## 1 Value of the Data

• The provided dataset offer the first comprehensive set of TGM/GEM hourly-based measurements collected simultaneously across Italy. Its value consists in providing near-continuous measurements at high temporal resolution over 4 years (2020 – 2023). It represents a robust regional/national baseline for atmospheric Hg levels. As data are collected from three Italian monitoring stations, representative of different geographical areas of the country, the provided dataset is useful in supporting a spatial analysis aimed at investigating atmospheric Hg variability and its relationship with natural and anthropogenic sources [1]. Other researchers, environmental agencies and policymakers can reuse this time series for the assessment of Hg pollution in Italy.
• An added value of this dataset is the integration of atmospheric Hg measurements with key meteorological parameters (e.g., wind speed and direction, temperature, relative humidity) recorded at each monitoring site through co-located sensors. The availability of the ancillary meteorological measurements enables the assessment of the physical processes influencing diurnal and seasonal variations. Hg data complemented with meteorological data is also essential for a source apportionment and dispersion analysis [2]. Researchers can utilize these merged time series to investigate the influence of atmospheric transport processes enhancing the ability to distinguish between local sources and long-range air mass transport influence.
• The inclusion of quality-controlled measurements of Gaseous Oxidized Mercury (GOM) and Particulate-bound Mercury (PBM) from a dedicated site provides insights into atmospheric Hg chemistry. These data can be reused by modelers for case studies aimed at improving the parameterization of Hg conversion processes (oxidation and reduction rates).
• The four-year time series (2020–2023) were collected following stringent operating protocols, aligned with the same standards employed within the Global Mercury Observation System (GMOS) network [3,4]. This standardized methodology ensures the reliability and comparability of the collected atmospheric Hg datasets.
• The dataset herein provided offers a robust base to evaluate the effectiveness of emission reduction strategies established by the international Minamata Convention on Mercury. Environmental agencies and policymakers can therefore reuse this dataset with the aim to verify policy compliance by tracking temporal trends and quantifying the progress made in line with Hg control targets [5].

## 2 Background

The original motivation for compiling this dataset derives from EU Directive 2008/50/CE (Ambient Air Quality Directive) and its Italian transposition (Legislative Decree 155/2010), which mandated the inclusion of systematic atmospheric Hg measurements within the national air quality monitoring network, specifically requiring at least three rural background stations. To address these European requirements, a dedicated collaboration agreement named ``Reti Speciali'' was signed in 2010, with the coordination of the Italian Ministry of Environment and Energy Security (MASE) [1]. This agreement established the network infrastructure and the methodological framework for monitoring atmospheric Hg levels across Italy and collecting related data. Procedures for data sampling, collection and managing adhere to the established reference methods and SOPs employed within the GMOS network [3,4].

Secondly, atmospheric Hg monitoring addresses Italy’s requirement for evidence-based data regarding the status and trends of this neurotoxic pollutant. Hg monitoring and control are regulated by the Minamata Convention on Mercury, an international framework to which Italy became a Party in 2021 [5].

## 3 Experimental Design, Materials and Methods

The monitoring activities were conducted in strict accordance with the European standard UNI EN 15852:2010 ("*Ambient air quality – Standard method for the determination of total gaseous mercury*'') [6]. This standard defines the performance characteristics and the minimum Quality Assurance/Quality Control (QA/QC) requirements for the continuous measurement of atmospheric Hg concentrations. Specifically, UNI EN 15852:2010 mandates the use of automated systems based on gold amalgamation followed by Cold Vapour Atomic Fluorescence Spectroscopy (CVAFS) or Atomic Absorption Spectroscopy (AAS).

The sampling and measurement protocols adopted by the "Reti Speciali'' network were detailed in a technical document produced by the associated Scientific Technical Committee (CTS) and outlined in Sprovieri et al. [7]. This national protocol was drafted by harmonizing the Global Mercury Observation System (GMOS) Standard Operating Procedures (SOPs) [8]. This alignment ensures that the Italian network meets the highest international scientific standards while adhering to the specific mandates of the European Air Quality Directive (2008/50/EC) and its Italian transposition (D.Lgs. 155/2010). Comprehensive details are provided in the associated research article [1].

In adherence to the sampling and measurement protocols, the experimental design of the database provided herein is defined by the specific Hg species analysed, as well as the selection of the three representative monitoring stations within the Hg network, as detailed below.

### 3.1 Definition of Atmospheric Mercury Species

Atmospheric Hg exists in three primary inorganic species:

• **GEM** (**Gaseous Elemental Mercury**): the predominant (95 % - 99 %) and the most volatile form of atmospheric mercury, allowing long-range transport;
• **PBM** (**Particulate Bound Mercury**): reactive mercury that is adsorbed to or contained within airborne particulate matter; and
• **GOM** (**Gaseous Oxidized Mercury**): gaseous reactive, and short-lived mercury that is highly susceptible to wet and dry deposition.

Depending on the method of measurement, another operatively defined atmospheric Hg species is referred to as:

• **TGM** (**Total Gaseous Mercury**): defined as the sum of all gaseous mercury species (TGM = GEM + GOM). In most environments, GOM concentrations are typically three orders of magnitude lower than those of TGM. Therefore, as widely supported by environmental studies [9], GOM contribute negligibly to the total gaseous Hg concentrations, making TGM measurements approximately equal to GEM.

### 3.2 Location of monitoring stations

Three dedicated monitoring sites were selected by the "Reti Speciali'' Scientific Technical Committee (CTS), as follow:

1. **Schivenoglia (SVG):** Rural background station in the Po Valley (45.02° N, 11.08° E, 16 m a.s.l.). Designated as a **Secondary station** for indicative measurements of **TGM** in ambient air;
2. **Montelibretti (MLI):** Suburban background station (42.11° N, 12.64° E, 30 m a.s.l.). Designated as a **Master station** aimed to perform atmospheric Hg speciation measurements, including **GOM and PBM**, in addition to GEM;
3. **Monte Sant'Angelo (MSA):** Rural background/coastal station on the Gargano ridge (41.67° N, 15.95° E, 30 m a.s.l.). Designated as a **Secondary station** for indicative measurements of **GEM**.

### 3.3 Measurements of atmospheric Hg in ambient air

In compliance with standardized methods, the network utilized two primary analytical techniques to ensure robust, continuous, and comparable monitoring of atmospheric Hg. In accordance with GMOS SOPs, the experimental design features three distinct instrumental approaches for ambient air Hg measurements, the primary characteristics of which are summarized in Table 1.

**Table 1:** Summary of instrumentation, analytical techniques, and detection limits for atmospheric Hg measurements.

Station	Parameter Measured	Instrument Model	Technique	Sampling Frequency/Time	Detection Limit
**SVG**	TGM	Tekran 2537X	CVAFS	5 min (1 L/min flow)	0.1 ng/m^3^
**MLI**	GEM, GOM, PBM	Tekran 2537X + 1130/1135 modules	CVAFS (Speciation)	GEM: 5 min (1 L/min flow). GOM/PBM: 3-hourly composite (2 h sampling, 10 L/min flow).	GEM: 0.1 ng/m^3^ GOM/PBM: 0.42 pg/m^3^
**MSA**	GEM	Lumex RA-915	AAS (Zeeman Effect)	5 min (7–10 L/min flow); 60-minute averaging interval	0.5 ng/m^3^

The above-mentioned instrumental approaches present the following features:

• **CVAFS Technique (Tekran 2537X):** The analyzer employs an alternating dual gold cartridge amalgamation cycle for TGM measurement, followed by thermal desorption at 500 °C and quantification via Cold Vapour Atomic Fluorescence Spectroscopy (CVAFS) using ultra-high purity argon as the carrier gas [10];
• **Speciation Unit (Tekran 1130/1135):** These modules opportunely integrated with the main Tekran module (Tekran 2537X) allow to differentiate GOM and PBM in addition to GEM measurements [11]. In particular, the 1130 unit uses a KCl-coated denuder to capture GOM while the 1135 unit uses a quartz regenerable particulate filter to capture PBM;
• **AAS Technique (Lumex RA-915):** This instrument performs a direct continuous measurement of GEM concentrations in the airflow based on differential Atomic Absorption Spectroscopy (AAS) with the Zeeman effect without the need for gold pre-concentration [12].

### 3.4 Data quality management

The acquisition of all raw Hg data was facilitated by a dedicated GMOS cyber-infrastructure described in Cinnirella et al. [13]. To ensure data quality, specific Quality Assurance (QA) and Quality Control (QC) procedures were performed using the GMOS-Data Quality Management System (G-DQM). This platform is a web-based system that performs automated data quality control in accordance with GMOS SOPs, as fully described by D’Amore et al [14].

Regarding the instrumentation employed, the following calibration specifications were also implemented to maintain measurement accuracy:

• Tekran Analyzers: Instrument performance was maintained through automated internal calibrations every 72 h using a permeation source [8,16];
• Lumex Analyzer: The RA-915AM was automatically calibrated every 48 h using a built-in cell saturated with Hg vapor [15,19].

## 4 Data Description

Quality-controlled data provided with this article consist of 4 distinct CSV files that collectively represent the baseline database for the Italian National Atmospheric Mercury (Hg) Monitoring Network [1].

The validated datasets were generated using the G-DQM system, the operability of which is described in detail by D’Amore et al [14]. In these records, TGM/GEM hourly averages and PBM/GOM 3-hourly averages are classified as valid only when data coverage reaches a minimum threshold of 75 % for the sampling interval.

To facilitate reproducibility, the raw measurements are provided in a zip archive (**rawdata.zip**) alongside the four validated datasets. This archive contains the original source files as automatically generated by the instrumentation. All raw data are provided as plain text files, organized into sub-folders for each of the three involved monitoring stations. Details regarding the raw data formats and internal instrument codes are available in the corresponding Tekran (Models 2537, 1130, and 1135) and Lumex (Model RA-915AM) technical manuals [[16], [17], [18], [19]].

Regarding the four validated datasets, they have been organized according to the atmospheric Hg species definitions previously described. Each of the four data files is identified by the acronym of the monitoring station from which the observations were collected (SVG/MLI/MSA). An overview of the provided files and their content is given at Table 2.

**Table 2:** Overview of the 4 provided data files and their specification content.

Data File	Station	Mercury Variable(s)	Time Resolution	Temporal Coverage	Co-located Data
SVG_TGM_Meteo.csv	**Schivenoglia (SVG)** Northern Italy	**TGM** (ng/m^3^)	Hourly	2020–2023	**Full Meteo**⁎
MSA_GEM_Meteo.csv	**Monte Sant'Angelo (MSA)** Southern Italy	**GEM** (ng/m^3^)	Hourly	2020–2023	**Full Meteo**⁎
MLI_GEM_Meteo.csv	**Montelibretti (MLI)** Central Italy	**GEM** (ng/m^3^)	Hourly	2020–2023	**Full Meteo**⁎
MLI_HgSpeciation.csv	**Montelibretti (MLI)** Central Italy	**GEM** (ng/m^3^) **PBM** (pg/m^3^) **GOM** (pg/m^3^)	3-Hourly	Limited Periods	None (Speciation data only)

⁎ **Full Meteo**: Air Temperature (T, °C), Wind Speed (WS, m/s), Wind Direction (WD, °), Relative Humidity (RH, %), and Rainfall (Precipitation, mm).

All four datasets are structured with the first column (**Column A**) dedicated to the timestamp of sampling collection, with the following features:

• **Timestamp Format:** YYYY-mm-dd HH:mm
• **Timestamp Zone: UTC** (Coordinated Universal Time)

In particular, three data files provide hourly atmospheric Hg concentrations (**TGM/GEM**) and corresponding meteorological data collected at the three involved monitoring stations. These three data files (**SVG_TGM_Meteo.csv, MSA_GEM_Meteo.csv, MLI_GEM_Meteo.csv**) are organized in a total of seven data fields/columns as summarized in Table 3. In detail, the first column (**Column A**) refers to the timestamp of sampling collection in the format above reported, the second column (**Column B**) contains **TGM** or **GEM** concentrations reported in ng/m^3^. The following columns (**Columns C – G**) contain meteorological information, specifically referring to: **Air Temperature** (T, °C), **Wind Speed** (WS, m/s), **Wind Direction** (WD, °), **Relative Humidity** (RH, %), and **Rainfall** (Rain, mm).

**Table 3:** Common structure of the three data files referring to TGM/GEM measurements and corresponding meteorological measurements (**SVG_TGM_Meteo.csv, MSA_GEM_Meteo.csv, MLI_GEM_Meteo.csv**).

Column	Data Field	Parameter Description	Format/Unit
A	**date**	Timestamp of sampling collection	YYYY-mm-dd HH:mm (UTC)
B	**TGM/GEM**	Concentration of Total Gaseous Mercury (TGM) or Gaseous Elemental Mercury (GEM)	ng/m^3^
C	**T**	Air Temperature	°C
D	**WS**	Wind Speed	m/s
E	**WD**	Wind Direction	Degree (°)
F	**RH**	Relative Humidity	%
G	**Rain**	Precipitation	mm

As Montelibretti (MLI) is the **Master Station** for speciation studies, a fourth file is included at the linked repository (**MLI_HgSpeciation.csv**). This dataset contains full atmospheric Hg speciation measurements, including PBM and GOM. For comprehensive analysis, the GEM values have been averaged to match the 3-hourly sampling period of PBM and GOM and made it available in the same data file. Due to the sampling frequency settings, the temporal resolution is lower (3-hourly). Due to the higher complexity of these integrated measurements the overall coverage is restricted to limited periods with available valid speciation measurements. The reason is primarily due to the rigorous QA/QC protocols of the G-DQM system, which invalidated periods affected by denuder breakthrough or filter saturation. The additional datafile referring to Hg speciation at MLI station is organized in a total of four data fields/columns (**Columns A – D**), as highlighted in Table 4.

**Table 4:** Structure of the data file referring to Hg speciation measurements performed at MLI station (**MLI_HgSpeciation.csv**).

Column	Data Field	Parameter Description	Format/Unit
A	**date**	Timestamp of sampling collection	YYYY-mm-dd HH:mm (UTC)
B	**GEM**	Concentration of Gaseous Elemental Mercury (GEM)	ng/m^3^
C	PBM	Concentration of Particulate Bound Mercury (PBM)	pg/m^3^
D	**GOM**	Concentration of Gaseous Oxidized Mercury (GEM)	pg/m^3^

## Limitations

The primary limitations of the provided datasets are concern the spatial coverage and the temporal availability of Hg speciation measurements, which is restricted to specific periods.

The national Hg monitoring network, in fact, currently consists of only three monitoring stations (Schivenoglia, Montelibretti, and Monte Sant'Angelo). While these sites provide a first baseline overview of atmospheric Hg concentrations across Northern, Central, and Southern Italy, this limited number of stations restricts the overall spatial representativeness of data. A more detailed characterization of atmospheric Hg across the geographically complex Italian peninsula would benefit from the inclusion of additional monitoring stations. An in-depth spatial analysis, supported by a higher density of observational points, would support a better investigation into source-related influences on atmospheric Hg levels.

Furthermore, atmospheric Hg speciation (including GOM and PBM) was performed only at the Montelibretti (MLI) Master Station. Due to the operational complexity and maintenance requirements of the Tekran integrated modules (Tekran 2537/1130/1135) the valid data coverage for the **MLI_HgSpeciation.csv** file is restricted to two shorter periods (2020–07–10 to 2020–12–14 and 2022–05–03 to 2023–05–07). Therefore, unlike the TGM/GEM datasets, the limited coverage of GOM and PBM time series restricts the possibility of identifying their seasonal trends and inter-annual variations.

## Ethics Statement

The authors have read and follow the ethical requirements for publication in Data in Brief and confirming that the current work does not involve human subjects, animal experiments, or any data collected from social media platforms.

## CRediT Author Statement

**Mariantonia Bencardino**: Writing – review & editing, Writing – original draft, Validation, Investigation, Formal analysis, Data curation, Conceptualization. **Antonella Tassone**: Writing – review & editing, Investigation. **Maria Martino**: Investigation. **Francesco D’Amore**: Writing – review & editing, Software, Data curation, Investigation. **Teresa Sprovieri**: Investigation. **Carmine Ungaro**: Investigation. **Virginia Andreoli**: Investigation. **Giulio Esposito**: Methodology, Investigation, Data curation. **Giorgio Siliprandi**: Investigation, Data curation. **Guido Lanzani**: Investigation, Data curation. **Lorenzo Angiuli**: Investigation, Data curation. **Alessandra Nocioni**: Investigation, Data curation**. Cristina Leonardi**: Supervision, Project administration. **Francesca Sprovieri**: Supervision, Resources, Formal analysis. **Nicola Pirrone**: Supervision, Funding acquisition.

## Declaration of generative AI and AI-assisted technologies in the manuscript preparation process

During the preparation of this work the author(s) used Gemini as assistant tool in order to check the English grammar and improve readability. After using this tool/service, the author(s) reviewed and edited the content as needed and take(s) full responsibility for the content of the publication

## Data Availability

ZENODOAtmospheric Mercury Data from the Italian Reti Speciali National Monitoring Network (2020–2023) (Original data)
